# Systematic study on nail plate assessment: differences in nail plate shape, thickness, power Doppler signal and scanning approach

**DOI:** 10.1007/s00403-022-02404-5

**Published:** 2022-10-21

**Authors:** Francesco Bellinato, Paolo Gisondi, Emilio Filippucci, Francesca Tozzi, Angelo Fassio, Giovanni Adami, Luca Idolazzi

**Affiliations:** 1 Section of Dermatology and Venereology, Department of Medicine, University of Verona, Verona, Italy; 2 Clinica Reumatologica, Università Politecnica Delle Marche, Ospedale “Carlo Urbani”, Jesi, Ancona, Italy; 3 Section of Rheumatology, Department of Medicine, University of Verona, Verona, Italy

**Keywords:** Ultrasound, Nail, Healthy, High frequency

## Abstract

Ultrasonography (US) of the nail is raising interest in the last years and its feasibility, quickness and amount of descriptive data may provide valuable information. Different authors presented several scanning approaches to nail complex in different pathological conditions, such as psoriasis, but no scanning protocol was ever proposed using healthy subjects as population of reference. The aim of the study was to establish a protocol for the US of nail plate and to assess whether the measurement of the nail plate is influenced by longitudinal vs transverse scan, sex, digit and hand dominance. Using high frequency probe and a Canon Aplio i800 machine, ultrasonographers took scans of nail plates of the hands from healthy subjects. Nail plate shape, thickness and power Doppler signal (PDUS) were evaluated and scans were taken both on longitudinal and transverse axis, at distal, middle and proximal portion of the nail plate or at a fixed angles of − 45°, 0° or + 45°. All the images were then revised and scored using a DICOM software, in order to allow good standards of accuracy and reproducibility. A total of 27 subjects (14 females and 13 males) were assessed. The measures did not result to differ in different portions or angles. Furthermore, no difference appears in sex or dominant vs not dominant hand. A decreasing and significant trend for nail plate thickness was found from the first to the fifth finger. Doppler signal was found in all but one subjects, with a range from almost absent to very evident. No difference was found between groups regarding PDUS. The data provided suggest that a proper scan protocol should include all the nails and evaluation should be done both on longitudinal and transverse axis. Since Doppler signal is highly variable in healthy subjects, its presence should be carefully considered as pathological finding. Observations provided by this study clarify important points of the scanning technique and solve doubts related to which nails should be scanned and where to evaluate quantitative parameters.

## Introduction

In the last years, a raising interest became evident for study of nail unit with ultrasound (US). First studies started before 2010 and were a point of interest especially in psoriasis and psoriatic arthritis [1–3]. After 2010, with improvement of probe technology, different approaches were used to study nail unit, with the aim of clarify non-specific or inconclusive clinical evaluation or fond subclinical changes that may not be evident but still important in diagnosis or differential diagnosis [4, 5]. US provide important details for a precise characterization of the nail disorders and is a non-invasive and extensively accessible technique that allows the evaluation of each one of the nail unit components with gray scale and also the assessment of vascularity with power Doppler technique [6] or with spectral Doppler evaluation of the Resistivity Index (RI) [7, 8]. The nail apparatus contains tissues of different echogenicity, and it is thus well suited for US examination [1]. Nail complex is composed by superficial structures very well described by US. The resolution power of the US imaging well illustrates the concept of sono-histology. This term describes the potential of US approach to dermatological field, as stated by *Ricci and colleagues* for soft tissues [9]. The exploration of potential also in the “hard tissue” such as the nails, is a valuable addition to which is already known about soft tissues [10]. Since ultrasonography is an operator-dependent method, it is generally required an appropriate training. Different nail unit disorders, such as psoriasis, lichen planus, onychomycosis, cysts and nail and skin tumors can be assessed in the diagnosis and follow-up [6]. The US of the nail plate is a technique that dates back since many years [2], but there is no agreement in the scanning protocol of this anatomical unit and only in the recent years a standard in definitions and terminology was proposed [11]. Some authors considered only few fingernails and the elements of interest [12–14], others applied US to all the fingernails and adjacent structures, such as distal interphalangeal joint (DIP) and enthesis of extensor tendon of the finger [15–17]. A recent systematic literature review [18] highlighted the differences in studies regarding nail plate. Furthermore, no data related to quantitative measures of healthy subjects are present in literature. Even when the scanning technique is only a qualitative analysis of the image, it requires a more stringent standardization in order to achieve a wide use in clinical practice and it could be integrated as for several skin conditions [19]. The introduction of very high frequency probes [20] and the advantages of the US applied to nail evaluation [21, 22] pushed this need quite far over in these years. Furthermore, the chance to explore the link between nail and enthesis in diseases, such as psoriasis and psoriatic arthritis, may be a strong determinant for development of US applied to nail, since several studies reports interesting but not univocal interpretation [23, 24]. Another future perspective is the understanding of Doppler signal role in the evaluation of the nail vascularization, especially at nail bed. No accordance was reached related to its role in revealing specific pathologic aspects or which is the technique to use for the best approach to vascular evaluation, power Doppler or spectral Doppler [7, 15, 25].

## Materials and methods

### Study design and subjects evaluation

The aim of the study is to establish a protocol for the US of nail plate. Secondly to assess whether the measurement of the nail plate is influenced by longitudinal vs transverse scan, sex, digit and hand dominance. Since the nail plate thickness can be measured in different sections, the distal, middle or proximal portion of every finger were taken into account. All the subjects were healthy men and women from the healthcare team of Dermatologic Clinic, University of Verona and measures were obtained in dedicated session to this task.

A brief medical history was collected before scanning to exclude any potential confounding alteration of the nail plate. Exclusion criteria were the presence of any pathological condition, taking any drugs and body mass index (BMI) out of the normal range (i.e. BMI ≤ 18.5 or BMI ≥ 25). Subjects were also asked for traumatic events of the fingers in the last 30 days and the subjects with traumatic working or sport activities were excluded. All the ones with a positive family history for any condition, such as psoriasis, which could affect nails were excluded.

### Ultrasound procedure

**Fig. 1:**
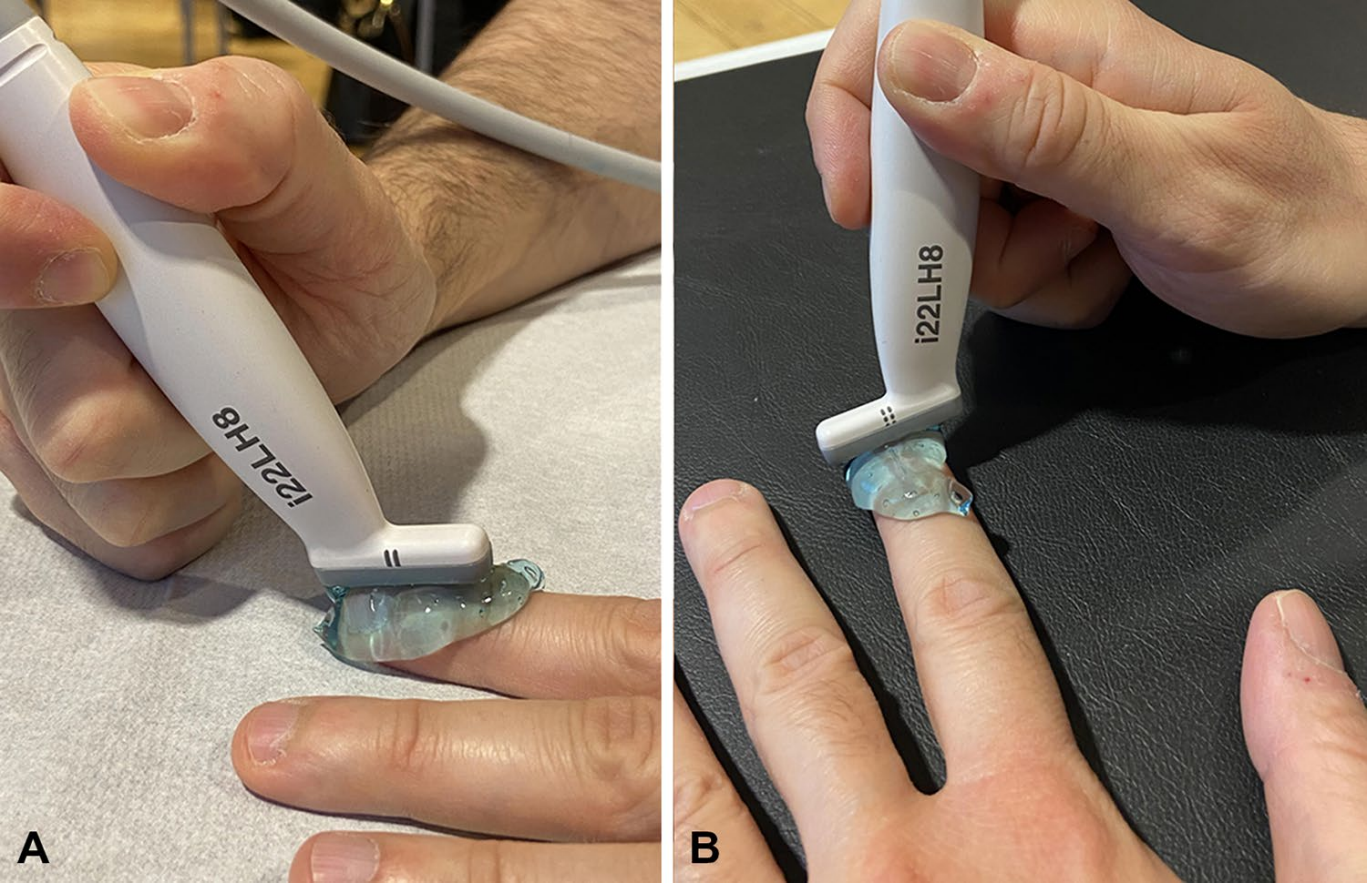
Position of the probe for longitudinal and transverse view. In **A** the probe is gently positioned in the longitudinal middle axis of the finger. A consistent amount of gel is place above the nail and then the probe is placed, avoiding compression. As showed, there is no direct contact between the probe and the nail plate, avoiding compression. Placing ultrasound gel is crucial and an adequate amount should exceed over the probe edges, as showed in **A** and **B**. In **B**, the probe is placed at the middle portion of the nail, to an angle of 90° of longitudinal middle axis. Also in this case the gel placement allow a full cover of the nail reducing artifacts and potential errors in the measurements

**Fig. 2:**
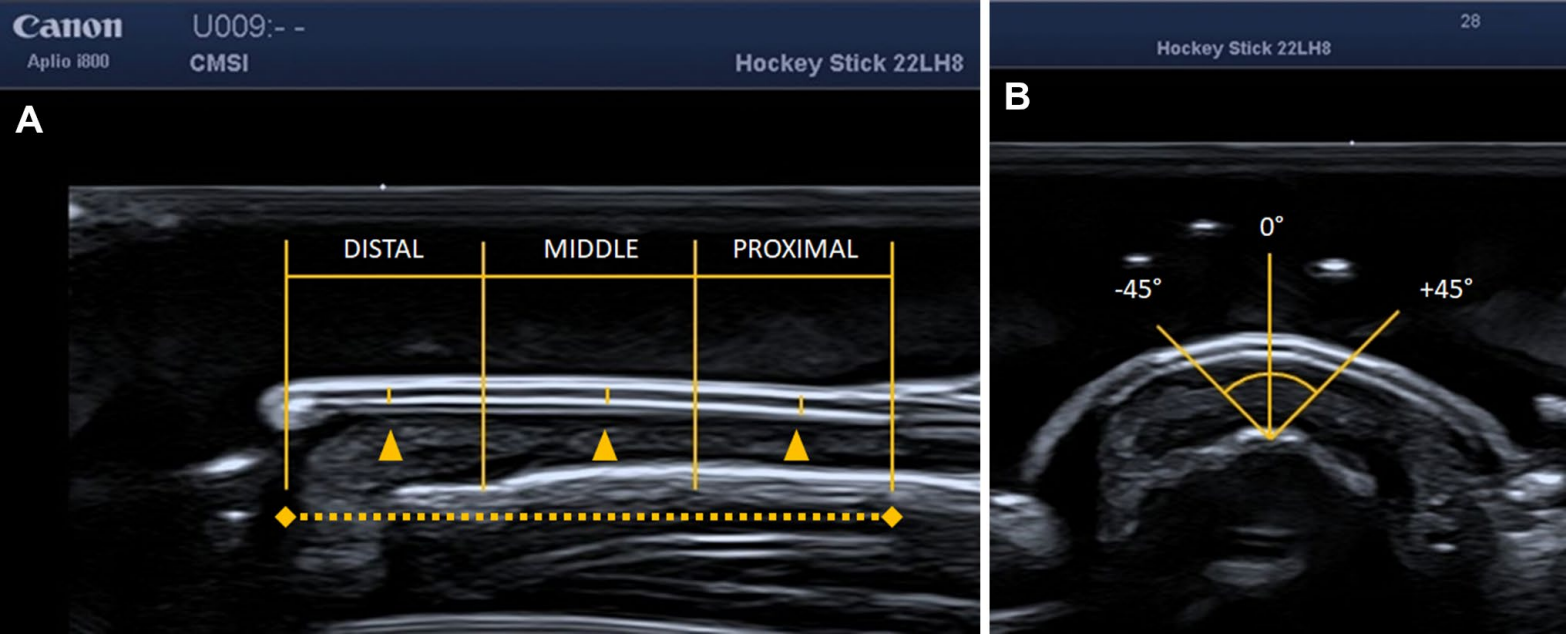
US appearance of the nail unit and points of reference for measurements. Below a consistent layer of gel (strongly hypoechoic), the first structure encountered was the sharp and hyperechoic band of the nail plate (dorsal lamina), then an anechoic part represents the intermediate lamina and the third lamina was the hyperechoic band (ventral layer of the nail plate). Then the nail bed appears as a hypoechoic space between the ventral layer of the nail plate and the hyperechoic cortical bone (area with arrowheads). The proximal longitudinal third stops at the eponychium. The nail matrix below the eponychium, a hypoechoic area with frequent Doppler signal is not shown in the figure. In picture **A**, the dotted line is the whole length of the nail considered for partition. This length was then divided in to three sections: distal, middle, and proximal. Every portion was then divided in half and at the middle the measurement of nail thickness was evaluated (arrowheads). In picture **B**, the transverse scan is marked by the angle tool of Weasis. Once the point of reference is obtained, the measurement was carried out carefully maintaining a 90° angle between the virtual ruler and the trilaminar structure of the plate

Two ultrasonographers independently performed the evaluations. A reliability exercise was conducted on selected images, with a *K* of Cohen of 0.82 between the ultrasonographers. After collecting clinical history, the subject was taken into a darkened room and prepared for US. The evaluation was conducted using strict standards, such as controlled room temperature and equal waiting rest time for all the subjects. They wait for about 20 min in a room with a standard temperature of 24 °C. The subject moved to another room with same standard temperature and sits in front of the operator, with hands prone and in resting position. The probe was placed over a consistent amount of gel, in order to avoid compression of the anatomical structures below and not squeeze blood vessels (as showed in Figs. 1A, 2). Sonographers checked also for bubbles artifacts and, if present, the gel was swiped and replaced, in order to avoid air scattering and ring artifacts. A longitudinal scan was performed, with the probe in the middle of the nail plate. The transverse scan was performed at middle portion of the nail (Fig. 1). The ultrasound examination was conducted with a Canon Aplio i800 machine with a multifrequency linear probe with setting at 22 MHz. This is the highest frequency allowed by the machine and fulfills the setting requested by the guidelines for performing dermatologic ultrasound examinations by the DERMUS Group [26]. Depth on the screen and the presets were kept constant through all the exam and did not change for any subject. Since Aplio i800 doesn’t rely upon the standard of PRF for sampling power Doppler, the machine setting was 0.8 cm/s with 12 MHz for frequency. All the images were stored and then analyzed using Weasis Medical Viewer (University of Geneva, Switzerland) [27]. The image was considered satisfactory if all the normal elements of the nail unit were clearly visible (Fig. 2).

The nail plate thickness was measured magnifying the image with the DICOM viewer software to achieve the highest accuracy for caliper placement. Every image was then processed as it follows. For the longitudinal scan, every nail plate was measured from the tip of the nail to the end of the visible part of the plate, then the ultrasonographer divided it in three equal portions. Then, the operator measured the thickness in the middle of each portion. For transverse scan, the ultrasonographers used reference points for angles provided by the software caliper and using these ones, they evaluated thickness at −45°, 0° and + 45° (Fig. 2). This was done for giving specific attention in maintaining the caliper perpendicular to the plate. These precautions descripted above are mainly aimed to ensure a proper and accurate evaluation of the three layers of the nail plate, avoiding potential artifacts. These are well known [28] and they occur especially for an incorrect position of the probe. Reverberation is most common and the main risk is to lose a sharp depiction of the trilaminar nail structure and changing of the histological interface in between adjacent layers when the acoustic interface is not perpendicular to the structure. In order to reduce the influence of this artifact the calipers for measures are placed at the near end of the first hyperechoic band and at the near end of the second hyperechoic band. This procedure should avoid the mistake of measuring a portion that potentially may vary in thickness, even if it is more frequent for hyaline cartilage [29]. Anisotropy may be another one, even if the signals are rarely reflected away from the transducer, since the angle of the beam doesn’t change very much. Also ring down artifacts due to bubbles were strongly limited since the scanning protocol was aimed to reduce them to the minimum, as stated above. Power Doppler was graded 0 if absent, 1 (mild) if the PDUS is present in less than 25% of the nail bed, 2 (moderate) if PDUS covers up to 50% of the area or 3 (severe), if it is greater than 50% with a semiquantitative scoring system widely used for this kind of evaluation [30].

### Statistical analysis

Considering the previous data available in the literature [2, 31–34], a sample of at least 20 subjects was considered adequate for a power of 0.8. All statistical tests were performed using SPSS Version 20 (SPSS, Inc., Chicago, IL, USA). This evaluation is consistent with other studies in literature [25, 35, 36]. The normality of continuous variables was assessed by a Shapiro–Wilk test. *T* test for independent samples or analysis of variance (ANOVA) were used to compare the means of the variables of interest between two or more independent groups, respectively. Multivariate ANOVA was used to explore the source of variability in nail plate thickness amongst covariates. The *p* value was considered significant if < 0.05.

All procedures were performed in accordance with the ethical standards of the responsible committee on human experimentation (institutional or regional) and with the Declaration of Helsinki of 1975, as revised in 1983.

## Results

After screening of 35 subjects, 27 (14 females and 13 males), mean age 31.43 ± 4.19 years were included. A total of 25 were right-handed and two left-handed. The mean measures are reported in Table 1. The qualitative analysis of the nail plate did not reveal any alteration of trilaminar normal structure. All the subjects showed nails with a very well-defined plate, without any grade of alteration. No difference between the measures of the proximal, middle, and distal portions of the same nail plate were found. Similarly, no difference between the transverse measures taken at different angles could be detected. At last, no difference between the longitudinal and the transverse mean measure of the nail plate thickness for each finger were found.

**Table 1:** Mean values of each nail plate thickness

	Proximal	Middle	Distal	Mean
Right longitudinal I	0.49 ± 0.05	0.49 ± 0.04	0.48 ± 0.05	**0.48 ± 0.01**
Right longitudinal II	0.48 ± 0.04	0.47 ± 0.04	0.47 ± 0.05	**0.47 ± 0.01**
Right longitudinal III	0.47 ± 0.04	0.47 ± 0.04	0.46 ± 0.05	**0.47 ± 0.01**
Right longitudinal IV	0.46 ± 0.03	0.45 ± 0.034	0.44 ± 0.04	**0.45 ± 0.01**
Right longitudinal V	0.43 ± 0.05	0.43 ± 0.04	0.41 ± 0.04	**0.42 ± 0.01**
Left longitudinal I	0.48 ± 0.045	0.48 ± 0.04	0.47 ± 0.04	**0.47 ± 0.01**
Left longitudinal II	0.47 ± 0.04	0.46 ± 0.05	0.46 ± 0.04	**0.46 ± 0.01**
Left longitudinal III	0.46 ± 0.04	0.46 ± 0.04	0.45 ± 0.03	**0.45 ± 0.01**
Left longitudinal IV	0.46 ± 0.03	0.45 ± 0.04	0.45 ± 0.03	**0.45 ± 0.01**
Left longitudinal V	0.42 ± 0.04	0.41 ± 0.04	0.40 ± 0.04	**0.41 ± 0.01**

	− 45°	0°	+ 45°	Mean
Right transverse I	0.47 ± 0.11	0.46 ± 0.11	0.46 ± 0.11	**0.48 ± 0.01**
Right transverse II	0.44 ± 0.10	0.44 ± 0.10	0.45 ± 0.11	**0.45 ± 0.01**
Right transverse III	0.43 ± 0.10	0.42 ± 0.09	0.44 ± 0.11	**0.44 ± 0.01**
Right transverse IV	0.40 ± 0.09	0.40 ± 0.09	0.42 ± 0.10	**0.42 ± 0.01**
Right transverse V	0.39 ± 0.09	0.39 ± 0.09	0.39 ± 0.09	**0.40 ± 0.01**
Left transverse I	0.47 ± 0.11	0.46 ± 0.10	0.49 ± 0.12	**0.48 ± 0.01**
Left transverse II	0.44 ± 0.01	0.42 ± 0.09	0.44 ± 0.10	**0.45 ± 0.01**
Left transverse III	0.43 ± 0.10	0.42 ± 0.01	0.42 ± 0.10	**0.44 ± 0.01**
Left transverse IV	0.40 ± 0.09	0.40 ± 0.09	0.41 ± 0.09	**0.42 ± 0.01**
Left transverse V	0.38 ± 0.08	0.38 ± 0.09	0.38 ± 0.09	**0.39 ± 0.01**

They are expressed in millimeters, as mean ± standard deviation. A quite evident decreasing trend is present from First to fifth nail plate of both hands. The mean values were taken at longitudinal middle portion and transverse 0° portion of nail plate

**Fig. 3:**
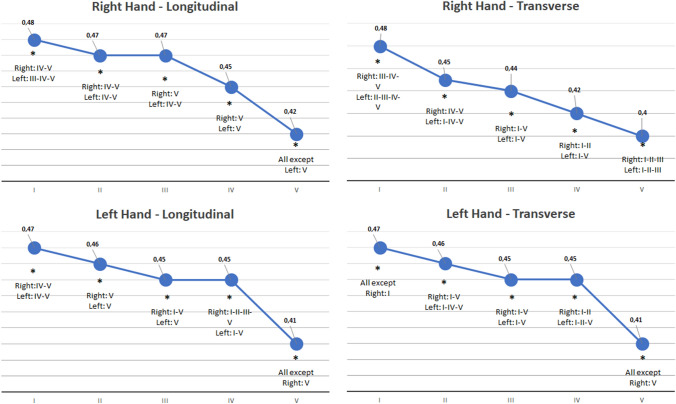
ANOVA test for comparing nail plate thickness among different nails. At the bottom line of every graph the corresponding nail of the hand in the graph title is reported with roman numerals. If an asterix (*) is present, it shows that the thickness of that nail plate significantly differs from all the others. Below the asterix are reported the nail plates of the fingers (both left or right) that differs from the corresponding one. *P* is considered for values < 0.05

When the mean value of the nail plate thickness of each finger is compared to the others, a statistically significant difference was found. The significance of *p* values < 0.05 is summarized in Fig. 3. As expected, a decreasing trend in thickness is evident from first to fifth finger of each hand and this occurs in the same way for right and left hand. Highest variability was found for the nail plate of the fifth finger.

**Fig. 4:**
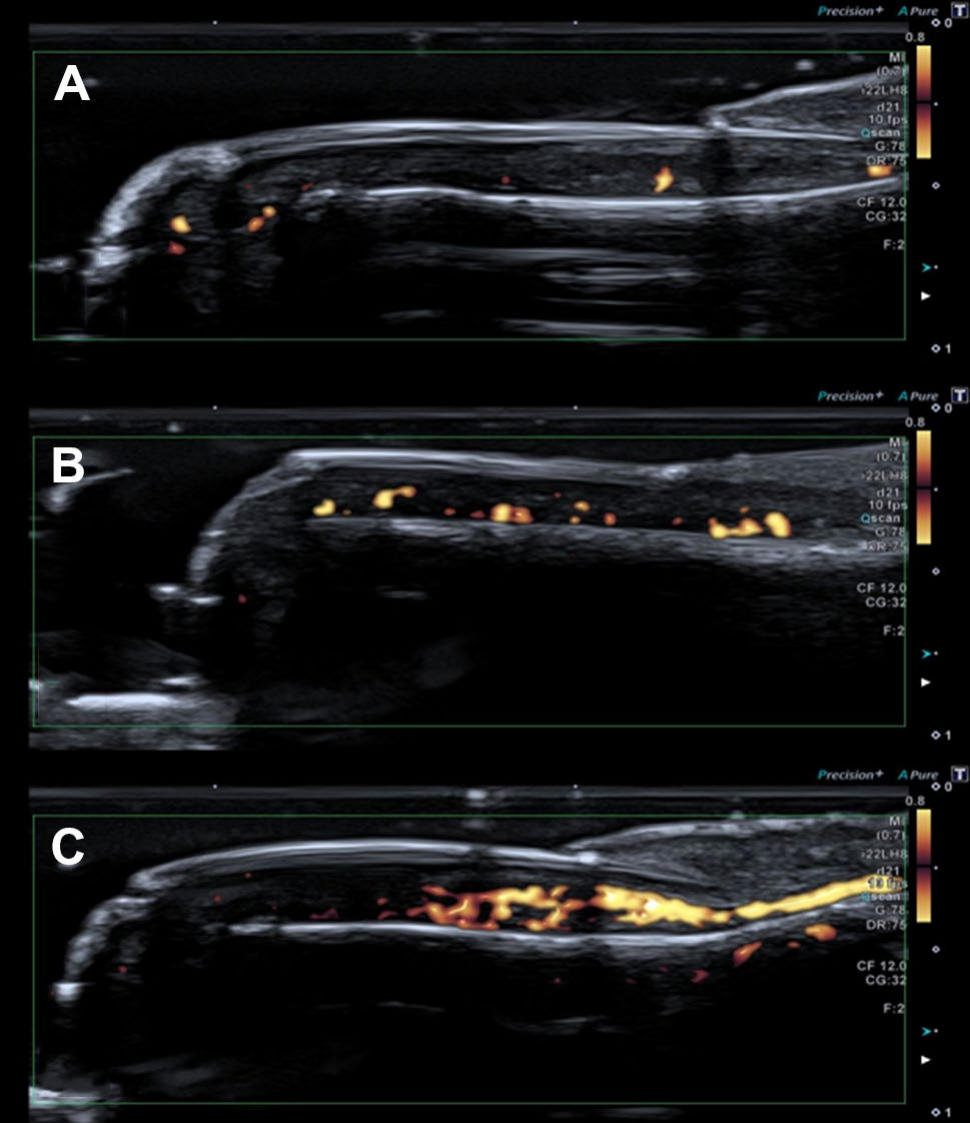
Power Doppler signal (PDUS) grading, accordingly with the classification of Gutierrez and colleagues [30]. In picture **A** the score is considered mild, with PDUS extension inferior of 25% of the total area of the nail bed. Two single spots and one confluent are clearly visible. In picture **B** several single and confluent spots are present, with moderate grade and an interested area covering less than 50% of the total. In picture **C** large and confluent vessels covered more than 50% of the area, configuring the PDUS as severe. Vascularization of the nail may be puzzling since three orders of vascular arcades (superficial, proximal and distal) creates anastomoses and may affect PDUS in magnitude also in healthy subjects because the vascular bed is rich in vessels

Regarding sex, mean measures of the nail plates were compared, but only a slight difference was found and only for the transverse scans of the nail plates of the first and second finger, at left hand (respectively *p* = 0.027 and 0.015) (Table 2).

**Table 2:** *P* values for comparison between males and females

Right longitudinal I	0.059	Right transverse I	0.079
Right longitudinal II	0.245	Right transverse II	0.051
Right longitudinal III	0.187	Right transverse III	0.163
Right longitudinal IV	0.846	Right transverse IV	0.727
Right longitudinal V	0.776	Right transverse V	0.255
Left longitudinal I	0.097	Left transverse I	0.027*
Left longitudinal II	0.247	Left transverse II	0.015*
Left longitudinal III	0.943	Left transverse III	0.180
Left longitudinal IV	0.663	Left transverse IV	0.051
Left longitudinal V	0.077	Left transverse V	0.200

Measurements were done at longitudinal middle portion and transverse 0° portion of nail plate

Power Doppler was found in all the fingers without a specific pattern of presentation, grading, or distribution (Fig. 4). Grading ranged from almost absent to almost present in all nail beds and was equally distributed in frequency of presentation and no statistical difference was observed.

## Discussion

In the present study, we suggest a simple protocol for the US of nail plate. One of the major findings is the decreasing thickness from first to fifth nail plate of each hand. This is very important since it answers to a critical question regarding which finger you must scan or how many nail plates you must consider for a proper evaluation. The evidence suggests that all the nails should be evaluated. The observed decrease suggests that a quantitative alteration of one nail plate may not be the same when found in different fingers. One example may be the alterations due to psoriasis, especially when they are not clinically evident [33]. Table 1 provides the mean values of all the fingers of left and right hand. The standard deviation of these measures is very low, and the sample size of this study is sufficient to provide credible quantitative values of nail plate thickness from a healthy population. It seems that scanning dominant or not dominant hand does not consist in major differences. One possible explanation of the decreasing in thickness is that the hand strength, size of the fingertips and tasks done by the different fingers during normal routine require thicker structures for balancing anatomical stress. This deduction leads to a second and important consideration. The fifth finger, both left and right, seems the one which is characterized by a wider variability. Unfortunately, not all the diseases affect all the fingers, and the authors suggest that a proper scanning protocol should involve all nails, avoiding potentials bias. Only nails affected by evident conditions (i.e. trauma, previous alterations for other disease like fungi, consistent top coat application) should not be evaluated.

Another frequent and important need is related to where positioning the probe during examination and which planes the ultrasonographer should adopt. No data in literature provide information for that. The authors chose to evaluate two different scanning approaches. The longitudinal scan (Fig. 1A) is the most traditional way of scanning nails. The nail plate is characterized by a variable curvature all along the structure. In some nails it is very evident, in other the plate is almost completely straight. Since minimal changes can interfere in the quantitative measurements, the need to understand if any portion of the nail is equal to another is mainly due to this fact. The beam angle and reverberation may cause artifacts and wrong positioning of the caliper. Since no difference was found in comparison between distal, middle, and proximal portion of the nail plate, the authors suggest the best section for measuring thickness is the middle portion. Here the curvature of the nail plate is minimal, such as the artifacts due to the reverberation. The same observation could be done for the transverse scan. Since minimal reverberation occurs at −45° and + 45° and tilting the probe does not assure any reduction in artifacts, authors suggest measuring the thickness at an angle of 0°.

The absence of difference between males and females makes the ultrasound of the nail more feasible in clinical practice. An open matter of discussion is the chance to include power Doppler in the evaluation of the nail. The Doppler signal presence was intended as a sign of inflammation [3, 37]. In this study we found the Doppler signal was almost always present. Furthermore, all the Doppler grades were found, and healthy nails showed high grade vascularization in some fingers, but none in adjacent ones of the same subject. This is not our exclusive finding and also *Aydin and colleagues* showed similar data [15]. Vascularity of nail bed in several disease may greatly vary and psoriasis is a good model for that, but seems that increased PDUS is not related to inflammation. The authors suggest to carefully consider the presence of Doppler signal as pathological finding. Since very different outcomes are present in literature, PDUS might be not the best approach for evaluating pathological conditions and spectral Doppler and RI could be a valuable alternative [7, 8, 25].

Limitations of the study are related to the relatively small study sample, composed by adult men and women. Another limitation is that we did not consider patients with manual work that may impact on the nail structure. This study does not provide any data about patients affected by diseases of the nail apparatus.

## Conclusions

This is the first study which make a proposal for a protocol to scan the nail plate and provides quantitative data about thickness in healthy nails. The authors suggest that all the nails should be scanned only in Brightness mode using gray scale. The scan should be taken at longitudinal middle section and at 0° of transverse section of the nail plate. If a standard is created, future evaluation for feasibility and reproducibility must be done. A more comprehensive comparison of nails of all the fingers should be done using also a population affected by specific diseases, but the observations provided by this study clarify important points of the scanning technique and solve doubts related to which nails should be scanned and where to evaluate quantitative parameters.
