# Correction to: Systematic study on nail plate assessment: differences in nail plate shape, thickness, power Doppler signal and scanning approach

**DOI:** 10.1007/s00403-022-02474-5

**Published:** 2022-11-25

**Authors:** Francesco Bellinato, Paolo Gisondi, Emilio Filippucci, Francesca Tozzi, Angelo Fassio, Giovanni Adami, Luca Idolazzi

**Affiliations:** 1 Section of Dermatology and Venereology, Department of Medicine, University of Verona, Verona, Italy; 2 Clinica Reumatologica, Università Politecnica Delle Marche, Ospedale “Carlo Urbani”, Jesi, Ancona, Italy; 3 Section of Rheumatology, Department of Medicine, University of Verona, Verona, Italy

**Correction to: Archives of Dermatological Research** 10.1007/s00403-022-02404-5

In the original version of this article, the given and family names of all the authors were incorrectly structured. The names were displayed correctly in all versions at the time of publication.

The original article has been corrected.

